# Supplementary material for: Are carbon emissions trading and green financial instruments synergistic? -Comprehensive quantitative research based on content analysis
Source: PLoS One. 2024 Mar 7;19(3):e0298601. doi: 10.1371/journal.pone.0298601 (PMC10919601; doi:10.1371/journal.pone.0298601)
Supplement: S1 Data — (ZIP) [file pone.0298601.s001.zip › Data/地级市Z_F工作报告.zip!/╒π╜¡/ßΘ╓▌╩╨/└┤╫╘╣1⁄2╓┌║┼í╛┬φ┐╦╩2╛▌═°í┐_2018.docx]

各位代表：

　　现在，我代表衢州市人民政府，向大会报告政府工作，请予审议，并请市政协委员和其他列席人员提出意见。

　　一、2017年工作回顾

　　2017年是七届政府的开局之年，是奔跑、战斗的一年，也是收获、振奋的一年。在省委省政府和市委的正确领导下，我们深入学习贯彻习近平新时代中国特色社会主义思想，全面贯彻党的十九大精神，认真落实省第十四次党代会和市第七次党代会精神，重谋划、强执行、求实效，圆满完成了市七届人大一次会议确定的各项目标任务，为开创衢州发展新局面奠定了扎实基础。

　　一年来，我们聚焦经济转型、生态建设、民生保障，发展质量不断提升。

　　经济发展提质增效。全面推进供给侧结构性改革，着力稳增长、调结构、防风险，经济运行稳中有进、持续向好。全市地区生产总值增长7.3%，增幅为2014年以来新高。从增长结构看，服务业带动力进一步增强，在三次产业中的占比达到48.6%，创历史新高。从效益结构看，全市财政总收入增长13.8%，增幅位居全省第三。规模以上工业企业利税增长44.3%、利润增长56.8%，两项增幅均居全省第一。服务业税收增长22.3%、占税收总收入的53%，其中金融业税收增长29.8%。柑橘、油茶、畜牧业等农业主导产业转型提升步伐加快，现代家庭农场等新型主体迅速成长，放心农业品牌进一步打响，衢江区获评全国首批农产品质量安全示范县，龙游县入选全国首批畜牧业绿色发展示范县，江山市水稻亩产连续两年创浙江纪录。从动力结构看，消费、出口拉动力不断增强，全社会消费品零售总额与外贸出口总额增幅均居全省第一。投资结构优化、效率提升，服务业投资、重大产业投资、生态环保投资分别增长12.8%、13.5%、24.2%。

　　生态环境持续优化。纵深推进“五水共治”，深化落实河长制，提前半年实现全域剿灭劣Ⅴ类水，13个省控以上断面首次全部达到Ⅱ类水标准，出境水质量实现历史性突破。狠抓治气攻坚，全面完成钢铁、热电、水泥三大行业清洁排放改造，市区PM2.5浓度下降到42微克/立方米，是开展数据监测以来的最低值。以中央和省环保督察为契机，铁拳整治环境突出问题，关停园区外排污企业31家，全面解决乌溪江饮用水源历史遗留问题。强力推进“三改一拆”，拆违1512万平方米、“三改”4009万平方米，分别完成年度任务的201.6%和250.6%。深入推进“四边三化”，被评为全省“四边三化”行动优秀市。深化美丽乡村建设，“一县一带”创建、农村环境整治卓有成效，全省美丽乡村和农村精神文明建设现场会在江山市召开，农村生活污水治理、垃圾分类基本实现全覆盖，农村“赤膊墙”问题基本解决，被评为全国“厕所革命”先进市、省小城镇环境综合整治优秀市。

　　民生事业全面发展。全面落实以人民为中心的发展思想，认真做好各项民生保障工作，圆满完成十个方面民生实事。坚持就业优先，实施积极的就业政策，新增城镇就业4万人。聚焦富民增收，城镇居民和农村居民人均可支配收入分别增长9.4%和9.8%，增幅均居全省首位。大力推进全民参保，社会保障提质扩面。着力加强住房保障，棚户区改造安置房开工6335套、建成4977套。积极推进教育现代化，礼贤小学等7所中小学校和13所幼儿园建成使用，新高考首战告捷。启动健康衢州建设，深化医药卫生体制改革，浙大衢州医联体正式运行，常山医共体建设试点顺利，衢江区成功举办首届世界针灸康养大会。大力发展养老服务业，居家养老服务照料中心实现城乡社区全覆盖。推进基本公共文化服务标准化，新建农村文化礼堂107个。构建安全生产“1+10”责任体系和“三个一”应急体系，深入开展安全生产隐患大排查大整治，事故起数、死亡人数分别下降36.7%和25.1%。治危拆违、除险安居工作取得新进展，实现城镇D级危房100%解危，完成农村危房改造9512户，治理重大地质灾害隐患点47个、搬迁6595人。创新社会治理机制，系统推进平安衢州建设，“雪亮工程”成为全国智慧治理典范，乡镇“四个平台”建设全省领先，全市刑事案件、侵财案件、治安案件大幅下降，社会治安持续好转。圆满完成“护航十九大”等维稳安保任务，龙游县荣获全国平安建设先进县。国家安全、国防动员、人民防空、民族、宗教、外事、侨务、对台等工作得到加强，海关、邮政、气象、检验检疫、新闻出版、对口支援等工作扎实推进，民航、档案、地方志、妇女、儿童、老龄、残疾人等事业取得新进展，社会救助、社会福利、慈善、红十字等事业取得新成效。

　　一年来，我们聚焦交通先导、城市赋能、产业创新，发展动能不断积蓄。

　　交通先导有效突破。杭衢高铁项目成功获批，把许多“不可能”变为“可能”，杭衢两地的时空距离将缩短到41分钟，实现枢纽接枢纽、西站通西站、高地到高地、通达变通勤，“东融杭州、杭衢一体”成为可能，同城效应得以显现，为衢州发展打开无限空间。以杭衢高铁为基础，谋划推进高铁新城、杭深高铁近海内陆线和浙西航空物流枢纽三大战略性项目，展现出一张全新的发展蓝图。衢九铁路通车运行，衢宁铁路加快推进，铁路地区规划总图研究取得重要成果，确定了“米”字型铁路网架构，着力打造省内第二大铁路枢纽。衢江航运建成并具备通航条件，46省道樟廿公路顺利通车，客运班车实现“村村通”。美丽沿江公路、物流大道延伸工程等项目快速推进，龙游港区等重大项目开工建设，杭金衢高速拓宽、351国道衢州段实现节点开工，义金衢上高速、杭淳开高速、常山江航运开发、高铁西站综合客运枢纽等重大项目前期扎实推进，衢州现代综合交通运输体系正加快形成。

城市赋能谋篇开局。强化规划赋能，按照产城人文融合发展思路，打造市区核心圈层，启动高铁新城开发，谋划了由高铁小镇、教育小镇、医养小镇、快乐运动小镇、科创金融小镇、文创文旅小镇和儒学文化小镇组成的特色小镇群落，每个小镇突出一个主题功能，统筹“主题、配套、生态、商住、运动休闲”五大空间，致力打造新型城镇化的新样板。因为相信、所以看见，衢城百姓心气大增，衢商回归步伐加快，外地客商纷至沓来，20多个国家驻沪总领事带队来衢考察洽谈。强化项目赋能，统筹市区重大公建项目布局，启动四省边际中心医院、体育中心、便民服务和文化艺术中心等重大项目。全面推进城市建设“新十大专项”，信安湖景观桥、书院大桥、衢化西路、衢化东路、火车站片区路网等建成通行，信安湖喷泉投入使用，九华路过江隧道开工建设，盈川东路全线开工、市区内环线将形成闭环;礼贤片区基础设施、西安门大桥拓宽改造、三衢路绿化美化、城东污水厂扩容提升和海绵城市建设等加快推进，新城吾悦、万达广场、常山东方、开化金佰汇等一批城市综合体建成运营。强化人气赋能，开展国家运动健康城市创建试点，组织“畅想信安、寻美绿道”健步走等全民健身活动，举办第五届市运会、衢州全程马拉松、中国滑水巡回大奖赛等赛事，运动休闲成为衢州城市新标签。水亭门历史文化街区获中国人居环境范例奖和浙江文化创意街区称号，灯光秀让古城再成“网红”。城市创建捷报频传，入选联合国首批可持续发展示范城市，获得全国文明城市提名，柯城区、开化县成为全省示范文明区(县)，国家卫生城市创建实现市县“满堂红”。

产业创新探索新路。加快传统产业转型，推进“四换三名”“三强一制造”“两化”融合等工作，战略性新兴产业增加值增长13.4%，衢州高新园区在全省30个高新园区考核中名列第四，中铁轴承列入省特别重大产业项目。积极培育智慧产业，成功引进阿里巴巴大数据、中兴克拉物联网、安恒信息科技、北讯电信、网易电子商务、中铁五院浙江分院等高端项目和创新团队，开辟数字经济发展新路径。农村淘宝实现市域全覆盖，全市网络零售额增长62.2%。发展壮大幸福产业，积极创建国家全域旅游示范区，实施乡村休闲旅游三年行动计划，开展“全球免费游衢州”活动，旅游接待人数、旅游总收入分别增长21.4%和25.6%，江郎山·廿八都景区获评5A级景区，水亭门、红木小镇获评4A级景区，常山县获得“国际慢城”命名，大荫山入选国家首批体育特色小镇，柯城区获评全国休闲农业和乡村旅游示范县，江山市、开化县获评省全域旅游示范先进县。系统构筑创新平台，京沪杭深“科创飞地”成为吸引高端要素的新平台，创新大厦成为区域创新发展的新标杆，国家级高新园区成为新旧动能转换的新高地，新增2家国家级、2家省级众创空间，新设35个省级电商产业基地。整合完善创新政策，出台创新驱动加快绿色发展实施意见，发挥政府产业基金作用，整合建立科创大专项资金，有效实现工业和科技专项资金的整合、资金和基金的并轨、财政资金和社会资本的联动、市县之间的统筹。有效激活创新主体，全面落实人才激励措施，加大人才培育引进力度。实施科技企业“双倍增”行动计划，新认定国家高新技术企业57家、省级科技型中小企业196家。积极推动企业上市挂牌，新增主板上市企业2家、“新三板”挂牌企业9家。

　　一年来，我们聚焦改革创新、开放开发、实干担当，发展格局不断打开。

　　改革创新赢得主动。全力开展“最多跑一次”改革，以“一窗受理、集成服务”为重点的改革模式成为全省标杆。打破部门壁垒、打破信息孤岛，实现群众和企业办事“一窗受理、一套标准、一网通办、一站服务”，全省“最多跑一次”改革现场会在衢州召开。在此基础上，推动“最多跑一次”改革向中介机构、企事业单位和基层延伸，“多审合一”“测验合一”改革试点在全省率先突破，得到国家发改委首肯，“最多跑一次”实现率、满意率分别达到97.3%、98.7%，持续保持领先。全面创新政府运行机制，强化大联动、大统筹、大整合，突出决策、执行、监督“三位一体”，推进政府工作项目化、清单化、责任化，建立专班工作制，构建项目工作“4421”体系，政府运行更加有序高效。深入推进重点领域和关键环节改革，国家绿色金融改革创新试验区建设全面启动，钱江源国家公园试点区总体规划正式发布，“多规合一”、国家主体功能区等试点扎实推进;投融资体制改革步伐加快，PPP模式推广应用、国企改革重组、国资清查整合等工作稳步开展，市区机关事业单位办公用房搬盘整合成效显著。

　　开放开发推动跨越。坚持把区域开放作为最大的改革。强化理念引领、打开思维格局，从“衢州人的衢州”走向浙江的衢州、中国的衢州乃至世界的衢州，从“传统产业下的衢州”走向新经济、新产业下的衢州，从“原有交通条件下的衢州”走向现代大交通格局下的衢州，放开了眼界、放宽了心胸、放大了格局、放出了活力。强化优势再造、打开时空格局，全力打造杭衢山海协作升级版，与杭州建立了涵盖海创园、人才、旅游、体育等33个领域的战略合作关系，衢州将加快融入杭州都市圈、创新生态圈。阿里巴巴等一批新经济龙头企业汇聚衢州，这是衢州开放开发的初步红利，也是衢州扩大开放的重要动力。开放促创新、开放促转型、开放促发展成为普遍共识、形成强劲态势。

　　实干担当打造铁军。深刻剖析政府系统存在的十个方面问题，提出了新一届政府的“十条军规”，努力打造战斗的团队、奔跑的团队、温暖的团队。坚持严明军规、正风肃纪，突出治理“不作为、慢作为、乱作为”现象，倡导“结果第一”“无条件、有办法”“最多讲一次”，念好“叮、盯、钉”三字诀，落实市委市政府决策部署抓铁有痕、掷地有声。坚持勇于担当、攻坚克难，干成了一批多年想干而没有干的大事难事，直面问题调整市中心医院选址、在建大楼变身创新平台，垃圾焚烧发电项目破除“邻避效应”平稳开工，斗潭片区危旧房改造整体腾空拆除全面完成，市农贸城关停平稳有序，乌溪江饮用水水源地衢江牛头湾自然村整体搬迁。坚持惩恶扬善、弘扬正气，市县两级全面开展“树正气、打歪风”专项行动，推动了174个项目扫尾攻坚，新征土地1.3万亩，拆除房屋2754幢、79.5万平方米，依法处理涉案人员65人，铁拳治乱象、铁腕护发展。坚持奖罚分明、抓人促事，以干事为导向、以实绩论英雄，去年以来共提议市委记功嘉奖5批47家单位，批评问责25批48家单位，每季通报“十条军规”正反典型，政府队伍精气神得到极大提振，“最多跑一次”改革、新农村建设、公积金管理等工作在全省年度考核中排名第一，市公安局、规划局、环保局、市场监管局等16个部门单位在全省年度考核中被评为优秀，市安监局被国家安监总局授予全国先进单位。

　　各位代表，格局决定结局，谋划赢得未来。一年来，市政府以“谋划一个新时代、干出一片新天地、打造一个新政府、锻造一支新铁军”的决心，谋划衢州发展、推动政府工作。尤其是紧紧围绕建设“活力新衢州、美丽大花园”，编制形成了《衢州市大花园建设行动纲要》，明确今后一个时期重点落实打开大通道、建设大配套、提供大产品、提升大环境、深化大协作“五大任务”，加快一批改革创新示范、一批特色小镇、一批重大项目落地，打造幸福产业、航空物流、新能源三个“万亩空间、千亿量级”产业大平台。大花园为衢州带来了一个完全不一样的格局、完全不一样的未来，已成为全市上下共同的奋斗目标和美好愿景!

　　各位代表，过去一年成绩来之不易，这是“八八战略”在衢州生动实践的结果，是省委省政府和市委正确领导的结果，是全市人民团结一心、拼搏奉献的结果。在此，我谨代表市人民政府，向各个领域辛勤劳动的全市人民，向给予政府工作有力支持和监督的人大代表、政协委员，向各民主党派、工商联、各人民团体和社会各界人士，向驻衢部队官兵，向所有外来投资者和建设者，向所有关心和支持衢州发展的港澳同胞、台湾同胞、广大侨胞和海内外朋友，表示衷心的感谢和崇高的敬意!

　　同时，我们也清醒地认识到，衢州新一轮发展正处在谋篇布局的起步阶段，发展的内生动力和创新活力有待加强，新旧动能转换和产业转型升级任重道远;大花园建设中，特色高端目的地大产品尚未形成;教育、文化、体育、医疗、养老等民生事业发展与群众期望仍有差距，低收入群众增收任务仍然艰巨，生态环境、食品安全等领域治理任务仍然繁重;政府工作的观念理念、体制机制、方式方法还需要不断适应新时代新形势新要求，政府工作人员的担当意识、开放意识、创新意识、服务意识、协同意识亟待增强，谋划力、执行力有待提升。对此，我们一定高度重视，全力以赴，采取更加有力的措施加快解决。

　　二、总体要求

　　党的十九大开启了中国特色社会主义新时代，省第十四次党代会赋予了衢州建设大花园的新使命，互联网时代、高铁时代、消费升级时代为衢州转型发展带来了新机遇。大花园让衢州找到方向，杭衢高铁让衢州赢得未来，“最多跑一次”让衢州跑出活力，衢州已步入厚积薄发、裂变发展的新阶段，已站上属于自己的风口!共同的梦想，凝聚越来越多的“衢州力量”;宏伟的蓝图，增添越来越多的“衢州自信”，这是衢州加快发展的动力源泉。没有什么比全市上下对衢州未来发展充满坚定的必胜的信心，更为宝贵的资源和财富!

2018年是全面贯彻党的十九大精神的开局之年，是改革开放40周年，是决胜全面建成小康社会的关键之年。做好政府工作，既要坚持与时俱进，紧跟时代脚步，更要保持战略定力，一张蓝图绘到底，一任接着一任干，持续发力、久久为功、必有所成。当前及今后一个时期，政府工作的总体要求是：高举习近平新时代中国特色社会主义思想伟大旗帜，全面贯彻党的十九大、十九届二中三中全会和今年全国“两会”精神，坚定不移沿着“八八战略”指引的路子走下去，坚定不移把“绿水青山就是金山银山”理念践行下去，大力弘扬红船精神、浙江精神，紧紧围绕“活力新衢州、美丽大花园”，全面推进交通先导、城市赋能、产业创新三大战略举措，扎实开展“两山”实践示范区和绿色金融改革创新试验区创建，努力使衢州成为大湾区的战略节点、大花园的核心景区、大通道的浙西门户、大都市区的绿色卫城，全方位推进高质量发展，为成为全省经济发展新的增长点和新的特色亮点、与全省同步实现“两个高水平”奠定坚实基础。

　　具体工作中，重点把握好三方面要求：

　　(一)更大格局推动衢州高质量发展，加快融入“四大建设”。建设大湾区大花园大通道大都市区，是浙江省打造现代化先行区的战略举措，大湾区是空间特征、大花园是普遍形态、大通道是发展轴线、大都市区是发展极。融入“四大建设”，是衢州新一轮发展的重大机遇。全省“四大建设”，衢州一个都不能缺席!打造大湾区的战略节点，就是要从核心区、统筹区、联动区三个层面全面对接大湾区，把衢州打造成为杭州大湾区向内陆拓展的战略节点，浙江统筹杭州、台州、温州三个湾区的战略节点，杭州湾和粤港澳两大湾区联动式发展的战略节点。打造大花园的核心景区，就是要突出建设“大花园+大平台”“目的地+集散地”“重大交通基础设施+重大公共服务配套设施”，加快构筑“国家公园+美丽城市+美丽乡村+美丽田园”空间形态，争取实现5A级景区县(市、区)全覆盖，努力成为“诗画浙江”中国最佳旅游目的地和世界一流的生态旅游目的地。打造大通道的浙西门户，就是要加快构筑智能高效的现代综合交通网络和物流体系，全面接轨浙江创新大通道、开放大通道、海洋经济大通道和生态旅游大通道，使衢州真正成为四省边际中心城市和浙江内陆开放桥头堡。打造大都市区的绿色卫城，就是要深化杭衢协作，加强重大战略定位、重大规划、重大基础设施、重大产业布局、重大政策导向、重大资源要素的市域统筹，积极参与新一轮杭州都市圈规划建设，推进杭衢同城化一体化，成为杭州都市区的绿色卫城。

　　(二)更高要求推动衢州高质量发展，打造“四个升级版”。坚决打破观念障碍、思维定势、路径依赖、利益束缚，开拓新思维、开辟新道路，聚焦“四个升级版”，再造竞争新优势。打造绿色发展升级版。大花园是新时代衢州高质量发展的基本路径，是新发展理念、新发展模式、新发展要求的集中体现。要举全市之力打造“花园式环境+花园式产业+花园式治理”，加快传统产业弯道超车、新兴产业换道超车。打造“最多跑一次”改革升级版。推动“最多跑一次”改革由“一窗受理”向“无差别受理”提升，由公共服务向企业服务、中介服务、基层治理延伸，由事前行政审批向事中事后监管服务延伸，以“最多跑一次”改革引领推动各重点领域、关键环节的全面改革，实现大党建统领、大联动治理、大数据应用、大融合推进，提升区域治理体系和治理能力现代化水平。打造山海协作升级版。以创新合作为重点，推动山海协作从单向扶贫输血向双向合作造血转变、从传统产业梯度转移向创新成果转化落地转变、从市县分散单独协作向市域一体对口协作转变，走出新时期山海协作的新路子。打造特色小镇升级版。按照产城人文高度融合要求，从单个小镇建设，升级为小镇群落建设;从产业、文化、旅游、社区四大功能，升级为城市美好生活的新社区、产业创新服务的综合体、带动区域发展的新引擎。

　　(三)更实举措推动衢州高质量发展，统筹“四个两手抓”。聚焦高质量发展的重点领域和关键环节，统筹施策、精准发力，确保各项工作落到实处。在开放开发上，既要打开杭衢高铁有形大通道，也要打开山海协作升级版无形大通道，加快杭衢同城化、杭衢一体化。在产业培育上，既要打造美丽经济幸福产业，也要打造数字经济智慧产业，让美丽更智慧、智慧更美丽。在改革创新上，既要打破产业生态方面的“坛坛罐罐”，也要打破政务生态方面的“坛坛罐罐”，努力建设一个适应现代市场经济、现代民主法治、现代互联网条件的现代新型政府。在大花园建设上，既要打造城市大花园，也要打造乡村大花园，市域统筹、市县联动、城乡互动，合力建设一个美丽富饶的大花园。

　　三、2018年重点工作

　　2018年，是我市全面建设大花园的第一年。我们要以大花园为总战略总方向总目标，全面实施大花园建设行动纲要，全面落实大花园建设2018年攻坚任务书。新一年，我市经济社会发展的主要预期目标为：地区生产总值增长7%左右;城镇新增就业2.2万人以上，城镇调查失业率控制在5%以内，城镇登记失业率控制在3.5%以内;一般公共预算收入增长7%;城镇居民和农村居民人均可支配收入分别增长8%和9%;全员劳动生产率增长8%左右;投资稳定增长，投资结构持续优化;节能减排降碳完成省定目标任务。重点抓好十项工作：

　　(一)大力推进重大改革创新。重大改革先行先试、抢抓红利持续领先，是活力新衢州的重要标志。深入推进“最多跑一次”改革，突出机制创新，推动“一窗受理”向“无差别受理”提升;深度推进政务数据整合，实施国家级政务信息系统整合共享应用示范工程，进一步打通部门之间、省市之间的信息孤岛，拓展政务服务大数据运用，实现审批留痕、监管有据，图纸入库、信息共享;深化企业投资项目审批制度改革，瞄准施工图审查、施工许可审批、企业用地审批等关键环节，全面推行“多审合一”“测验合一”“多评合一”“标准地”建设，打造企业投资项目审批流程最优的全国样板。以创促建实施一批试点示范创建项目。扎实推进全国“两山”实践创新基地、绿色金融改革创新试验区、钱江源国家公园体制试点、国家运动健康城市等国家级试点示范建设，加快新型智慧城市、现代基层治理体系建设示范市等重大改革创新，并努力转化为政策红利、项目支撑、资金支持和区域品牌。大力推进国资国企改革，完善公司法人治理机制，完成国企市场化转型，组建大金控、大城投、大交投，推动国有资本做强做大做优。做好新一轮机构改革的各项准备。

　　(二)全力打造山海协作升级版。牢牢把握全省实施新一轮山海协作的重大机遇，全面推动杭衢两地精准对接、高效合作、深度融合。以战略融入为统领，强化杭衢一体化顶层设计和政策机制，全方位对接杭州、融入杭州、借势杭州，打造杭州“后花园”，融入杭州都市经济圈、创新生态圈。以培育新动能为核心，聚焦创新领域的战略合作，积极引入“浙大系”科教资源，嫁接“阿里系”裂变基因，承接“大走廊”创新要素;开工建设衢州海创园二期，推进特色小镇群落与未来科技城、梦想小镇、云栖小镇的对接融通，大力引进科技人才、项目、资源;加快滨江高新园区衢州分园落地建设，共建衢州江干科创转化基地，加速杭州科技创新成果在衢州产业化。以条块协作为重点，推动杭衢两地形成“1+8”市域一体对口协作新模式，“1”即衢州对杭州，“8”即衢州6个县(市、区)对杭州县(市、区)、高铁新城对未来科技城、集聚区对大江东，提升战略合作的系统性、联动性、协同性;加大杭衢两地部门对接协作力度，推动“1+33”战略合作全面落地见效。以互利共赢为保障，加快杭衢两地基础设施互联互通、生态保护联防联控、公共服务共建共享，更大范围打通两地政策服务后台，实现杭衢同城效应。

　(三)构建外联内畅的现代综合交通运输体系。空铁公水齐头并进，打造国家区域交通枢纽城市，构筑智能高效、“承东启西、联南通北”的现代综合交通网络和物流体系。铁路，全线开工杭衢高铁项目，加快推进衢宁铁路建设，扎实做好杭深高铁近海内陆线、衢黄铁路等项目前期。公路，全面开工杭金衢高速拓宽、351国道衢州段、205国道开化段改建、江山市和常山县综合客运枢纽等重点项目，加快市综合客运枢纽项目建设，基本建成衢州沿江美丽公路，推进义金衢上、杭淳开高速等项目前期。水运，加快龙游港区、衢江港区建设，实现衢江航运一期4级航道正常运营，争取衢江航运二期常山江航运开发工程列入国家高等级内河航道规划，深化浙赣运河、江山江航运前期。民航，主动融入浙江民航强省战略，推进浙西航空物流枢纽项目前期，开通昆明、青岛等新航线，争取鄂尔多斯等城市航线。系统推进交通治堵，统筹内循环+外循环、动态交通+静态交通、路面管理+智慧治理，深化城市交通组织规划，推进智慧交通项目建设，新建和改造一批停车设施，优化提升公交服务，推广新能源汽车分时租赁系统，让城乡交通更顺畅、群众出行更便捷。

　　(四)加快建设现代品质之城。围绕城市赋能,致力打造产城人文高度融合的中心城市。聚焦高铁新城主平台，以新型城镇化理念为引领，全面启动特色小镇群落建设，体现自然生态、低碳环保、运动休闲、智慧治理，彰显宜居、宜业、宜文、宜游，打造具有衢州特色的现代新城。扎实推进老城有机更新，以“南孔圣地、衢州有礼”城市品牌为核心，启动南孔文化复兴工程，加快儒学文化小镇建设，加强古建筑保护和古街区开发，以产业振兴带动文化复兴，推动“南孔文化重重落地”，打造具有衢州特色的历史名城。加快市区核心圈层开发，以创建5A级景区为目标，加强“一江两岸”空间立体性、平面协调性、风貌整体性、文脉延续性的规划和管控，打造集聚城市能量的“台风眼”，实现古城新区交相辉映、历史现代各展风采。全面加强城市管理，以全国文明城市提名为新起点，开展文明城市创建新一轮“十大专项整治”，推进国际花园城市和可持续发展国际示范城市建设;立足新型智慧城市建设，加强城市精细化管理，让城市环境更宜居、百姓生活更舒适。全力推进城市项目建设，开工高铁新城路网水网、地下综合管廊、体育中心、便民服务和文化艺术中心、严家淤全民健身岛、半岛地下空间、市区路灯体系完善、十里江滨公园提升等工程，完成高铁新城整体连片征迁、新儿童公园、府东街改造、浙西大道改造、三衢路绿化美化、信安湖光影秀等工程，加快四省边际中心医院、九华路过江隧道、西安门大桥拓宽改造、盈川东路、水亭门街区、生活垃圾焚烧发电、衢江治理二期等工程，深化荷一路过江通道等项目前期，提升斗潭、衢化、礼贤街、火车站等片区基础设施配套。

　　(五)全力推进产业转型升级。围绕高质量发展要求，坚定不移推进产业创新，加快传统产业弯道超车、新兴产业换道超车，努力再造产业发展新优势。培育发展数字经济智慧产业。加快推进阿里巴巴大数据、中兴克拉物联网、安恒信息科技、网易电子商务等一批项目建设，争取在互联网、物联网、大数据、人工智能等智慧产业发展上取得新突破。实施新型智慧城市、城市数据大脑行动计划，加快数据港、信息港建设。推进省电子商务创新发展试点，推动电商产业持续快速发展，网络零售额增长40%以上。培育发展美丽经济幸福产业。把提供大产品作为重中之重，加大5A级景区培育提升力度，加快全民健身中心、森林运动小镇、石梁溪田园综合体、铜山源水库旅游综合开发、世界(衢江)食品安全创新示范基地、新田铺田园康养综合体等建设，推进红木小镇、“归江山居”颐养小镇、赏石小镇、根缘小镇等开发;办好国际铁人三项邀请赛、中国围棋冠军赛、中国滑水巡回大奖赛、衢州马拉松等赛事活动，积极筹办“一带一路”国际贸易合作论坛和2019年第四届全国智力运动会，培育一批高端精品民宿，持续开展“全球免费游衢州”活动，全面创建国家全域旅游示范区。推动传统产业改造提升。坚持质量第一、效益优先，深入推进化工、钢铁、建材、造纸、装备制造等重点传统行业的绿色化、智能化、高端化改造提升。大力发展军民融合产业。开展国家质量强市示范城市和国家知识产权示范城市创建，加快推进“三强一制造”“企业上云”“两化”融合。实施“凤凰行动”，支持优势企业上市、并购重组，“辅导一批、股改一批、签约一批”，新增主板上市企业1-2家，向证监会报会和辅导企业3家，签约后备企业7家，全市直接融资比重达到20%以上。加快构筑大创新体系。打通京沪杭深“创新飞地”、创新大厦、花园258、科创金融小镇、浙江中关村科技产业园、国家级高新园区等创新平台，集聚一批优质资源要素和高端项目人才，促进一批创新成果、创新项目在衢州转化落地。健全人才政策体系，加强人才服务保障，推进“技能衢州”建设，强化高端人才引进，打造优质高效的人才生态。支持企业加大科研投入，确保R&D经费支出占GDP比重达到1.5%以上。

　　(六)全面实施乡村振兴战略。按照“产业兴旺、生态宜居、乡风文明、治理有效、生活富裕”总要求，落实“五大任务”、推进“十大专项”，加快建设乡村大花园，努力成为全省实施乡村振兴战略的排头兵。致力深化农村改革，统筹推进农村产权制度改革，有效盘活农村闲置资产资源;优化农村创业创新环境，大力支持乡贤以项目回迁、资金回流、智力回乡、技术回援等多种形式回乡兴业，回报桑梓、反哺故土，引导更多城市能人下乡创业。致力发展农村产业，以农业供给侧结构性改革为主线，突出质量兴农、绿色兴农、科技兴农，大力发展“互联网+”农业、绿色现代农业，积极培育农业平台型创新型企业，创建省级农业绿色发展示范区、畜牧业绿色发展示范市，进一步打响“三衢味”等放心农产品品牌;突出农业全产业链和农业产业平台建设，大力引进培育新型农业主体，推进乡村资源与旅游、文化、体育、健康等融合发展，打造一批田园综合体和农业特色小镇。全面落实粮食安全责任制。致力提升农村环境，开展农村人居环境综合整治，强力推进农村“厕所革命”和“垃圾革命”，积极推进农村生活污水处理提标改造和运维提升，深化“一户多宅”整治;推进农村全域景区化，深化“一县一带”建设，做好村庄规划和农房设计，加强农房管控和风貌引导，整体推进美丽庭院、精品村、示范乡镇、风景线创建和小城镇整治，完善“四好农村公路”建管养机制，深化“一村万树”行动，争取新增A级景区村200个以上，创建3A级景区村20个以上。致力推动农民增收，多渠道促进农民收入持续普遍较快增长，启动新一轮低收入农户增收计划，加强精准帮扶，实现低收入农户共享小康，确保完成消除集体经济薄弱村任务。致力加强乡村治理，坚持自治、法治、德治相结合，构建“党建+智慧治理+共建共治共享”的乡村治理体系，推广“村情通+全民网格”模式;加强农村文化礼堂、文化广场等阵地建设，注重传统村落保护利用，开展移风易俗行动，形成文明乡风、良好家风、淳朴民风。

　　(七)全力打造中国最优营商环境城市。围绕“审批事项最少、办事效率最高、投资环境最优、企业获得感最强”要求，以列入全国营商环境评价试点市为契机，发挥优势抻长板、找准差距补短板，通过三到五年努力，全力打造中国最优营商环境城市。构建优质高效的政务环境。以深化“最多跑一次”改革为“牛鼻子”，强化“事前舍得放，事中事后主动服务敢于管”，持续推进简政放权、提高服务效能。坚持“服务零距离、监管不扰民”，当好服务企业的“店小二”，着力解决企业反映强烈的问题，建立“亲”“清”新型政商关系。构建富有活力的创业环境。建立财政“大专项”政策整合体系，强化精准扶持、协同发力，提高政策的有效性，助力创业创新，集聚高端要素。不折不扣落实各项企业减负措施，强化依法治税，探索建立全市收费目录清单，实现清单之外无收费，力争全年为企业减负降本50亿元以上。构建公平竞争的市场环境。高度关注中小企业发展，深化土地、金融、用能、排放等资源要素配置改革，更好发挥市场机制作用。打破地方保护、打破隐性壁垒，依法保障企业各方面公平待遇，营造公平、稳定、透明、诚信的市场环境。构建平安和谐的社会环境。深入开展扫黑除恶专项斗争，持续开展“树正气、打歪风”专项行动，全面开展护企促商专项整治，打造“全国社会治安首善之地”。严格落实安全生产“1+10”责任体系，强化隐患排查治理，确保事故起数、死亡人数和单位生产经营死亡率持续下降，坚决遏制重大事故发生。

(八)持续打好生态环境整治攻坚战。统筹推进治气、治水、治土，不断夯实生态底本。开展治气攻坚三年行动，打响蓝天保卫战，确保市区PM2.5浓度下降到40微克/立方米。狠抓主要污染源治理，巩固提升钢铁、水泥、化工等重点行业清洁排放改造成果，抓好施工扬尘、餐饮油烟、秸秆焚烧等治理管控，实施全市域城区烟花爆竹“双禁”工作。深化“智慧环保”建设，建立覆盖全市的负氧离子监测网络体系，定期发布清新空气评价信息。高标准推进“五水共治”，全面落实“河长制”，实施“碧水行动”计划，推进全流域综合治理，确保13个省控以上断面水质稳定达标。启动5万吨市污水处理厂改扩建工程，新增城镇污水管网建设165公里。各县(市、区)分别创建2条以上“美丽河流”和2个以上“污水零直排区”。推进龙游高坪桥水库等项目建设，加快开化水库项目前期。推进土壤污染防治，实施重点污染地块修复治理工程，建立覆盖城乡的生活垃圾分类处置体系。持续开展“四边三化”行动，打造一批省级精品项目。扎实推进“三改一拆”治危拆违专项整治行动，加快存量违建分类处置，严格管控新增违建，巩固扩大“无违建”创建成果。

　　(九)切实保障和改善民生。持续推进教育现代化。实施第三轮学前教育三年行动计划，新建、改造一批义务教育学校和幼儿园。深化推进职业教育产教融合、校企合作，启动浙江大学工程师学院衢州分院建设，建成阿里衢州学院、阿里衢职院等校企实训基地。加强师资队伍建设，提升教育核心竞争力，擦亮衢州教育品牌。加快健康衢州建设。深化公立医院体制改革，创新与高端医院合作模式，加快一批医联体、医共体试点项目建设。完善分级诊疗政策体系，健全医保差别化支付制度，努力缓解群众就医难、看病贵等问题。全面加强食品药品安全监管，保障人民群众饮食用药安全。大力发展文化事业。优化提升基本公共文化服务，深入实施文化惠民工程。加快发展文化产业，推进文创文旅小镇等平台建设，积极培育新业态，促进文化产业与旅游、体育、医养等融合发展。提高社会保障水平。全面落实就业创业政策，促进高校毕业生、就业困难人员等重点群体就业创业，鼓励外出务工人员返乡创业。深化低保提质扩面。推广医疗救助“一站式”结算，加大对残疾人、低收入居民等群体救助力度。优化城乡居家养老服务，提升养老服务质量水平。加强棚户区改造，建立覆盖面更广的住房保障体系。开展“无欠薪行动”，构建和谐劳动关系。

　　(十)努力建设人民满意的现代新型政府。以更高标准、更严要求、更实作风加强政府自身建设，努力打造现代新型政府，切实做到为人民着想、对人民负责、受人民监督、让人民满意。坚持为民勤政。深入践行以人民为中心的发展思想，牢记向人民作出的庄严承诺，真心倾听群众呼声，认真解决群众身边的“关键小事”，做到脑勤腿勤、主动作为，确保干一件、成一件、好一件。坚持高效施政。把强谋划、强执行作为政府工作的生命线，着力提高行政质量、行政效率和政府公信力。坚持课题研究先行，常态化制度化开展重点课题研究。以提升效能为导向，深化专班工作制，推进政府工作项目化、清单化、责任化，强化政府系统资源整合、力量打通、流程再造、数据共享，加快政府数字化转型。健全工作督查机制，突出“首办责任制”和“一把手负责制”，形成“督查协调再督查、协调督查再协调”的闭环，确保工作落到实处。坚持依法行政。严格遵守宪法法律，严格执行重大行政决策程序规定。认真落实市人大及其常委会各项决议决定，积极支持政协履行职能，全力办好人大代表议案建议和政协提案，自觉接受人大依法监督、政协民主监督以及监察、司法、审计和社会监督。全面推进政务公开，让权力在阳光下运行。坚持从严治政。讲政治、守规矩、顾大局，坚决维护以习近平同志为核心的党中央权威和集中统一领导，坚决落实省委省政府和市委决策部署。在市委的坚强领导下，做到“同心同德同向、合心合力合拍、尽心尽力尽责”，做到“同一话语体系、同一工作体系、同一责任体系”。严守“十条军规”，严惩“十不”现象，完善权责一致、奖罚分明的激励约束机制，健全鼓励担当、激励创新的容错免责机制。落实全面从严治党主体责任，切实加强政府廉政建设，严守中央“八项规定”，努力营造风清气正的政务生态。始终保持永不懈怠、拼搏奋斗的精神状态，不负使命、砥砺前行。

　　各位代表!以人民为中心，增进人民福祉，是政府一切工作的出发点和落脚点。今年，我们按照“群众提、大家定、政府办”的理念，梳理形成民生实事候选项目，提请本次大会票决选出其中10个。民生实事项目确定后，我们将保质保量完成，让人民群众有更多的安全感、获得感、幸福感。

　　各位代表!新时代充满希望，新机遇令人期待，新征程催人奋进。让我们更加紧密地团结在以习近平同志为核心的党中央周围，高举习近平新时代中国特色社会主义思想伟大旗帜，在市委的坚强领导下，不忘初心、和衷共济，实干担当、锐意进取，为加快建设“活力新衢州、美丽大花园”，成为全省经济发展新的增长点和新的特色亮点，与全省同步实现“两个高水平”而努力奋斗!
